# Safety analysis of new medications in clinical trials: a simulation study to assess the differences between cause-specific and subdistribution frameworks in the presence of competing events

**DOI:** 10.1186/s12874-023-01985-7

**Published:** 2023-07-13

**Authors:** Astrid Genet, Kathrin Bogner, Ralf Goertz, Sarah Böhme, Friedhelm Leverkus

**Affiliations:** 1 Pfizer Pharma GmbH, Linkstraße 10, Berlin, 10785 Germany; 2 AMS Advanced Medical Services GmbH, Am Exerzierplatz 2, Mannheim, 68167 Germany

**Keywords:** Competing risks, Drug development, Cause-specific versus subdistribution hazard ratio, Safety analysis, Simulation study

## Abstract

Safety is an essential part of the evaluation of new medications and competing risks that occur in most clinical trials are a well identified challenge in the analysis of adverse events. Two statistical frameworks exist to consider competing risks: the cause-specific and the subdistribution framework. To date, the application of the cause-specific framework is the standard practice in safety analyses. Here we analyze how the safety analysis results of new medications would be affected if instead of the cause-specific the subdistribution framework was chosen. We conducted a simulation study with 600 participants, equally allocated to verum and control groups and a 30 months follow-up period. Simulated trials were analyzed for safety in a competing risk (death) setting using both the cause-specific and subdistribution frameworks. Results show that comparing safety profiles in a subdistribution setting is always more pessimistic than in a cause-specific setting. For the group with the longest survival and a safety advantage in a cause-specific setting, the advantage either disappeared or a disadvantage was found in the subdistribution analysis setting. These observations are not contradictory but show different perspectives. To evaluate the safety of a new medication over its comparator, one needs to understand the origin of both the risks and the benefits associated with each therapy. These requirements are best met with a cause-specific framework. The subdistribution framework seems better suited for clinical prediction, and therefore more relevant for providers or payers, for example.

**Supplementary Information:**

The online version contains supplementary material available at 10.1186/s12874-023-01985-7.

## Background

Safety data are an essential part of the clinical evaluation of new medicinal products and regulatory submissions. However, their analysis might be challenged by the existence of competing risks. These are intercurrent events, defined as mutually exclusive events (death, other adverse events, change of treatment, noncompliance, end of study, etc.) whose occurrence precludes the event of interest from happening [1]. Competing risks are common. They are present in the vast majority of clinical trials [2, 3] and might bias the results [3, 4]. They represent a well-recognized problem in the analysis of adverse events [5, 6] and general recommendations urge the use of survival techniques that methodically account for the presence of competing risks [2, 5–7]. These techniques acknowledge that for a given adverse event there are other types of risks that occur at the same time.

**Fig. 1:**
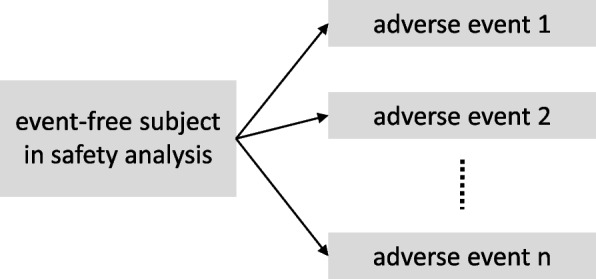
Graphical presentation of the Markov multi-state model in a competing risks setting

The standard survival data situation corresponds to a Markov process with the two states: “event-free” and “event”. Splitting the “event” state into more states corresponding to different causes (“event 1”, “event 2”, “dead”, etc.) results in a Markov model for competing risks [8]. The analytical object in the presence of competing risks is the same as in standard marginal survival analysis: to estimate the probabilities, also named risks, and hazard rates of the event of interest over time and, if relevant, to assess whether there are differences between groups. However, a competing risks setting that extends the capabilities of analysis of two state survival models to deal with multi-state models (cf. Fig. 1) is required, when subjects can experience more than one event [9]. The risk of the event of interest over time is estimated among the risk of other competing events whose occurrence precludes it from happening. The concepts of risks and rates generalize easily to the competing risk situation: hazard rates become cause-specific hazard rates and risks become cumulative incidences [10].

Two statistical frameworks exist to perform survival analysis in the presence of competing risks: the cause-specific and the subdistribution settings. All standard methods for survival data apply to the cause-specific setting [11, 12] which focuses on the cause-specific hazard function. This function estimates the probability of each type of event separately, right-censoring individuals at the time of the competing event, as well as for loss of follow-up, withdrawal, or at the end of the observation time. For the subdistribution setting, specific approaches were developed that based on the cumulative incidence function [13, 14]. This function focuses on the cumulative incidence (or “subdistribution”) from a particular cause and does not treat competing events as censored observations. Consequently, individuals remain at risk for the event of interest even after they experienced the competing risk. This contra intuitive representation is however necessary to predict the correct cumulative incidence functions [13].

These settings differ in their definitions. The aim of this study is to compare their properties and to recommend how to perform safety analyses in clinical research and regulatory submissions. We investigate whether systematic differences exist between the estimates obtained with each approach and define to what extent the interpretation of the results of survival analysis depends on the choice of one or the other setting. For both settings non-parametric approaches [4, 8, 14, 15] as well as regression models [13, 16] exist. Classical hazard-based methods for survival data apply when analyzing cause-specific hazards: Kaplan–Meier and Nelson-Aalen estimators as well as the Cox proportional hazards regression model. These methods, however, do not allow to draw inference for subdistribution functions of competing risks. Specific approaches were developed: the Aalen-Johansen estimator and the Fine and Grey model. This paper focuses on (semi-) parametric approaches: cause-specific (Cox regression) and subdistribution hazard regression (Fine & Gray model). Both offer two major advantages in comparison to the non-parametric approaches. First, they allow to adjust for covariates when assessing and comparing event probabilities over time and thus provide more insight into the mechanisms that lead to the occurrence of an event. Second, they allow to use a fitted model to make predictions (e.g., for certain attributes of the population under study).

In the Methods section, we provide a brief, nontechnical description of the cause-specific and subdistribution settings in survival analysis. Detailed technical descriptions can be found elsewhere [8, 13, 16, 17]. A short introduction to the non-parametric estimators can be found in the Additional file 1. To examine and to compare the properties of the cause-specific and subdistribution settings in survival analysis, a simulation study was conducted. It covers all possible practical outcomes: from superiority to inferiority of the medical intervention and from small to large effect sizes. In the Results section, we report the results of safety analyses performed on each simulated dataset with a cause-specific and a subdistribution setting. Finally, the practical interest of both approaches is discussed in terms of their implications and relevance for safety analyses of new medicinal products and regulatory submissions. We insist on the point that the appropriate choice of either cause-specific Cox models or Fine-Gray subdistribution hazard models depends on the precise question of interest to the researcher or stakeholder: their differences make them complementary rather than interchangeable: they represent different points of views and therefore answer different research questions.

## Methods

### Cause-specific hazard regression

The Cox proportional hazards model [18] is expressed by the hazard function $h\left(t\right)$ presented in Eq. 1. In this model, $h\left(t\right)$ is determined by a set of covariates and expressed as:

1 $$h(t)= {h}_{0}\left(t\right) \times {e}^{\beta \mathrm{^{\prime}}{\varvec{X}}}$$

where $$t$$ is the time, $${\varvec{X}}$$ is a vector of covariates, $$\beta \mathrm{^{\prime}}$$ is the vector of regression coefficients that measures the effect size of each covariate on the hazard and $${h}_{0}\left(t\right)$$ is the baseline hazard, under the assumption that all explanatory variables are either set to zero ($${\varvec{X}}$$=0) or represent average values. The quantities of interest in the Cox model are the hazard ratios (HR) $${e}^{{\beta }_{j}}$$, where j $$\in$$$$\left\{1, 2, \dots , c\right\}$$ represents the $$c$$ covariates considered in the analysis. HR are relative measures of an effect between different values taken by a covariate. They do not provide any information about the absolute risks. A very common covariate investigated in medical and pharmacological research is the group attribution. In this case, in a binary setting with two groups, the HR associated with this covariate is the ratio of the rates of occurrence of an event in both groups. A value equal to one indicates no differences between the groups, a value of less than one indicates a higher and a value of more than one a lower rate of occurrence in the reference group. The cause-specific Cox proportional hazard model is a natural extension of the standard Cox proportional hazard regression where a model is fitted separately to each cause-specific hazard by censoring all individuals who experienced one of the competing risks before the event of interest.

### Subdistribution hazard regression

While in the Cox model the hazard for the event of interest only depends on its own (cause-specific) hazard, Fine and Gray [13] proposed a model that expresses an instantaneous hazard function $h\left(t\right)$ by the cumulative (subdistribution) hazard function $F\left(t\right)$ that is described in Eq. 2. The subdistribution model contains an additive component and the instantaneous risk of occurrence of the event of interest $k$, ${\mathrm{F}}_{k}(t)$, depends on all cause-specific risks. It can be expressed as:

2 $${\mathrm{F}}_{k}(t)= {\mathrm{F}}_{0,\mathrm{k}}\left(\mathrm{t}\right) \times {\mathrm{e}}^{\sum_{i}{\upgamma }_{i}{\mathbf{X}}_{{\varvec{i}}}}$$

where $k$ is the event of interest while i $\in \left\{1, 2, \dots , n\right\}$ represents all the competing events (including $k$) considered in the analysis. Analogous to the expression of the Cox model presented in Eq. 1, $t$ is the time and, ${\varvec{X}}$ is a vector of covariates. Similar to $\beta$ in the Cox model, $\gamma$ is the vector of regression coefficients measuring the effect size of each covariate on the cumulative hazard. ${F}_{0, k}\left(t\right)$ is the baseline cumulative hazard, that is the cumulative hazard under ${\varvec{X}}$=0. The regression coefficients $\gamma$ can be interpreted in a similar way as the $\beta$ from a Cox model, except that they are relative measures of risk between the values of certain covariate, taking into account competing events occur that preclude the occurrence of the event of interest. This means that the size of the effect due to each competing event on the HR for the event of interest cannot be isolated. It should be noted that the model considers an extended risk set where individuals are still at risk for the event of interest even after they experienced the competing risk. Fine and Gray acknowledged that this is unnatural but necessary in order to get a model that correctly predicts cumulative incidence functions [13].

### Simulation study

A simulation study was conducted to investigate the differences in the results of safety analysis performed in presence of competing risks when the subdistribution setting is chosen instead of the standard cause-specific setting. Three possible outcomes were considered, i. e. (1) superiority of the verum group compared to the control group, (2) inferiority of the verum group compared to control group, and (3) equivalence between both groups.

As commonly done in biometrics, HR was estimated to compare the risk of occurrence of the adverse event of interest between the verum and control groups [19, 20]. HR in the cause-specific setting (HR_cs_) was fitted by a Cox regression model and in the subdistribution setting (HR_sd_) by a Fine and Gray model.

The following assumptions were made:

- Each study comprised 600 patients allocated into two study groups (verum and control) in a 1:1 ratio.
- Two competing event types (the adverse event of interest and death as competing risk) were simulated with event times for both types following an exponential distribution. We selected a common and simple one-parameter event time distribution that implies a time constant hazard rate $h$(t)= $h$ that makes it easy to control the characteristics of the simulated data [21]. The hazard rates of the exponential distributions were defined according to the targeted median time to event ${t}_{\mathrm{0,5}}$ for a given treatment group and a given event type:
  $$h=\frac{log(2)}{{t}_{\mathrm{0,5}}}$$
- Administrative censoring occurred after 30 months if neither the primary event (adverse event of interest) nor the competing event (death) had occurred in a patient by then.
- The characteristics of the distribution of the competing event death were kept constant across all simulated scenarios. Median survival was set to 20 and 10 months for the verum and control groups, respectively, which corresponds to a HR of 0.5, in favor of the verum group.
- Median time to first adverse event was incremented in 1-month intervals between 1 and 20 months in the verum and control groups resulting in 400 patterns (i. e. 20 × 20), hereafter referred to as “conditions of interest”.

The following statistics were reported for the competing risk analysis of each condition of interest:

- Median time to adverse event (AE), median time to death and their corresponding standard deviations;
- HR_cs_ and HR_sd_, their corresponding 95% confidence intervals and two-sided *p*-values to investigate for group differences.

**Fig. 2:**
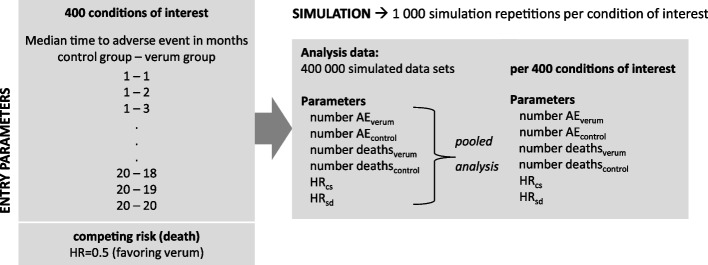
Overview of the simulation study procedure, the input parameters, and estimated parameters

The simulation of each of these conditions of interest was repeated 1 000 times. This value was chosen as a reasonable trade-off between the required accuracy of the results and the available computational resources. The statistics were separately assessed on each of the 1 000 datasets generated for each condition on interest and then pooled together according to Rubin’s rule (cf. Fig. 2) [22].

### Presentation of results

For an initial assessment of whether changing the setting from cause-specific to subdistribution leads to a change in the three possible outcomes of the safety analysis performed in a competing risk setting, results were classified into nine possible categories. These categories were defined in a two-step process:

First, starting from a cause-specific setting, the results of survival analysis of the 400 conditions of interest were classified into the possible outcome categories:

- Superiority if HR_cs_ < 1 and *p*-value ≤ 0.05
- Inferiority if HR_cs_ > 1 and *p*-value ≤ 0.05
- Equivalence if HR_cs_*p*-value > 0.05

Second, for each condition of interest, we assessed whether HR_sd_ fell in the same outcome category as the HR_cs_ or in one of the two possible alternative categories. This resulted in nine possible outcome categories when switching from the cause-specific to the subdistribution setting. The proportion of the 400 conditions of interest falling in each of these nine possible categories was reported.

Heat maps provide a graphical overview of the results, from the classification of the 400 conditions of interest to the observed differences between the true HR and the outcomes of the survival analysis performed in both the cause-specific and subdistribution settings.

### Software

All analyses were conducted using R version 3.6.1 [23]. The R package *survival* was used to fit the Cox proportional hazards model [17, 24]. The R package *cmprsk* was used to fit the Fine and Gray model [25]. The pooled-analysis of the parameters of the 1 000 simulated repetitions for each of the 400 condition of interest was done by the R package *mice* [26]. Heat maps for the graphical presentation of results were created with the R package *ggplot2* [27]. Detailed information on how to use these R packages can be found in the original publication for each package.

## Results

**Fig. 3:**
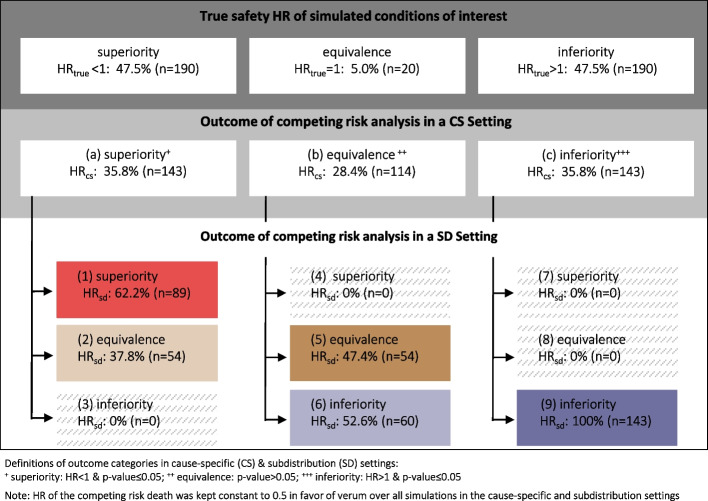
Classification of results of competing risk analyses in cause-specific and subdistribution settings into outcome categories

The true value of the HR (HR_true_) for safety analysis, defined as the entry value given to simulate the occurrence of safety events, is known. The HR of the competing risk death was kept constant to 0.5 in favor of verum over all simulations. Each condition of interest was categorized according to HR_true_: superiority (HR_true_ < 1), inferiority (HR_true_ > 1) or equivalence (HR_true_ = 1) of the verum group compared to the control group. In 47.5% of the simulated conditions of interest, verum was safer than control (superiority). The amount of the conditions of interest where verum was less safe than control (inferiority) was the same. In the remaining 5% of simulated conditions of interest, verum and control were equally safe (equivalence) (cf. Fig. 3).

Estimated competing risks HR_cs_ of the 400 conditions of interest ranged from 0.05 to 20.16 and HR_sd_ from 0.15 to 11.36.

As is detailed in Fig. 3, 35.8% of the competing risks safety analysis performed in a cause-specific setting resulted in superiority, 35.8% in inferiority and 28.4% in equivalence of the verum group compared to the control group. The slight differences between HR_cs_ and HR_true_ can be easily explained. Unlike HR_true_, HR_cs_ is calculated in a competitive risks setting where patients who experienced the competing event (death) before the event of interest were censored. Censoring leads to a reduced number of patients, especially towards the end of the observation period which may as well reduce the statistical power needed to detect existing differences between the treatment groups.

Among the conditions of interest that show superiority of verum in a cause-specific setting (*n* = 143), 62.2% still showed superiority when analyzed in a subdistribution setting (category 1). For the remaining 37.8%, the superiority of the verum group disappeared in the subdistribution setting. Statistical tests were not significant and the outcome category changed from superiority of verum to equivalence (category 2). A change from superiority of verum in the cause-specific setting to inferiority of verum in the subdistribution setting was not observed (category 3; cf. Fig. 3).

Of the conditions that showed equivalence between verum and control in a cause-specific setting (*n* = 114), none showed superiority when analyzed in a subdistribution setting (category 4). Equivalence between the two treatment groups remained in about half of the conditions of interest (47.4%; category 5) while the other half (52.6%, category 6) turned to inferiority of verum when analyzed in a subdistribution setting (cf. Fig. 3).

Among the conditions of interest that resulted in inferiority of verum in a cause-specific setting (*n* = 143), all remained significantly disadvantageous for verum (category 9) when analyzed in a subdistribution setting, the other possible outcomes of category 7 and category 8 were not observed in the study (cf. Fig. 3).

**Fig. 4:**
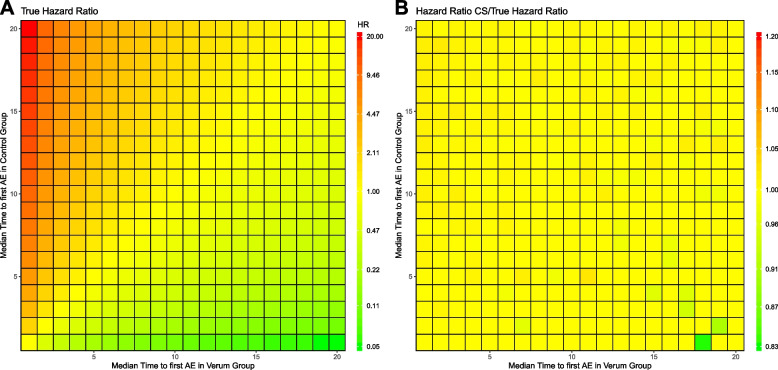
Heat map of **A** HR_true_ and **B** the ratio (HR_cs_/HR_true_). Legend: Each heat map in figure 4 plots the hazard ratios of all 400 conditions of interest of simulated safety analysis with competing risks by median time to first adverse event in verum and control groups

The heat map of panel (A) in Fig. 4 presents the HR_true_ of the entry values for the 400 conditions of interest in the simulated safety study with a constant HR of the competing risk (death) of 0.5 in favor of the verum group. The verum group is superior to the control group if the median time to first adverse event is longer than in the control group (HR_true_ < 1, green shadings, lower right part). Conversely, the verum group is inferior to the control group if the median time to first adverse event is shorter in the verum group than in the control group (HR_true_ > 1, red shadings, upper left part). If the median time to first adverse event is the same for both groups, they are considered equivalent (HR_true_ = 1, yellow shadings, the diagonal separating lower right and upper left parts). Figure 4, panel (B) shows for each condition of interest in the simulation study in the cause-specific setting the ratios of HR_cs_ and HR_true_. Accordingly, a ratio around the value of 1 (yellow shadings) indicates no difference between HR_cs_ and HR_true_. This is observed for most ratios of the 400 conditions of interest in the simulated safety study in a cause-specific setting; independent of median time to first adverse event in verum and control groups. For ratios with values less than 1 (green shadings), HR_cs_ is lower than HR_true_. This is observed for some conditions of interest, especially when the median time to first adverse event is much higher in the verum than in the control group. Only in these cases is a deviation in HR_cs_ observed in favor of the verum group. Ratios with values above 1 indicates higher HR_cs_ than HR_true_ values which is not observed in the simulated data.

**Fig. 5:**
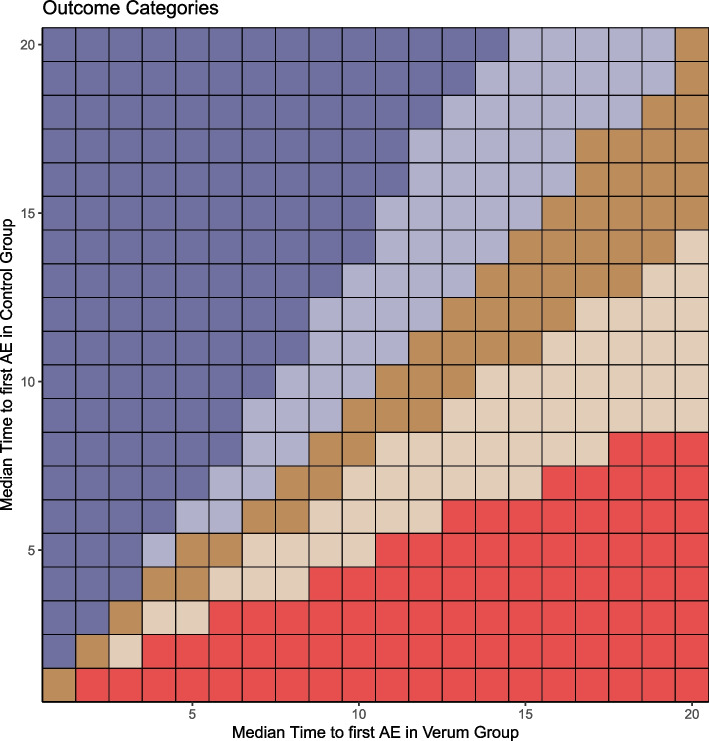
Map of outcome categories of safety analysis with competing risks in the subdistribution setting. Legend: Figure 5 plots all outcome categories of safety analyses for all 400 conditions of interest by median time to first adverse event in verum and control groups. The colors represent the outcome categories described in Fig. 3

Finally, the heat map in Fig. 5 indicates the categories into which the conditions of interest are classified according to the HR_sd_ from simulated safety analysis after switching from the cause-specific to the subdistribution setting (see also Fig. 3).

The safety analysis in the subdistribution setting, as in the cause-specific setting, resulted in superiority of the verum group over the control group if the median time to first adverse event in the control group is short and occurs earlier than in the verum group (red shading in Fig. 5; corresponds to category 1 in Fig. 3). However, when the median time to first adverse event in the control group increases, but is still shorter than in the verum group, results in the subdistribution setting no longer show superiority, but equivalence between both groups (light brown shading in Fig. 5; corresponds to category 2 in Fig. 3).

For the conditions of interest, for which in the cause-specific setting the safety in both groups was equal, the analysis in the subdistribution setting also shows equivalence, if the median time to first adverse event for both groups is close to each other (dark brown shading in Fig. 5; corresponds to category 5 in Fig. 3). If the median time to first adverse event however is earlier in the verum than in the control group, the outcome changes from equivalence in the cause-specific setting to inferiority of the verum group in the subdistribution setting (light blue shading, in Fig. 5; corresponds to category 6 in Fig. 3).

For all conditions of interest, for which in the cause-specific setting an inferiority of the verum group was the result of safety analysis, this is also confirmed in the subdistribution setting (dark blue shading in Fig. 5; corresponds to category 9 in Fig. 3).

All other possible outcome categories (3), (4), (7), and (8) of safety analysis when switching from cause-specific to subdistribution setting are not present in the simulation study data (see also Fig. 3).

In sum, the results of the simulated safety analysis with death as a competing event show that comparing safety profiles in a subdistribution setting is always more pessimistic than in a cause-specific setting. For the group with the longest survival and the safety advantage there is either no more advantage or a newly found disadvantage compared to its analysis in the cause-specific setting.

## Discussion 

### Understanding the etiology of risks for clinical evaluation

Defining the benefit/risk balance of medications in comparison to that of the standard of care in a given indication, implies to understand the origin of both the risks and the benefits associated with each therapy. The decision is based on acceptable trade-offs. Addressing epidemiological questions of etiology has long been recognized as the strength of the cause-specific setting [4, 12, 28–31], because of the censoring at the competing event. Censoring equals “disallowing” competing events so that censored patients could still experience the event of interest. Considering this hypothetical population, in which the event of interest would eventually happen for everyone, prevents competing events to get in the way when one is interested in comparing instantaneous rates of occurrence of the event of interest, between an intervention and its comparator. However, this hypothetical population may not be suitable for all research questions.

In the subdistribution setting, an extended risk set is considered where individuals remain even after they experienced the competing event. Fine and Gray themselves acknowledged that this approach is unnatural, since a patient who experienced the competing event of death is no longer at risk of developing an adverse event. The consequence of this construction is that causal parameters are not accurately estimated. But this extended risk set is necessary to correctly predicts cumulative incidence functions, particularly useful for prediction. In this sense, the method is best suited to predict clinical outcomes on patients at risk (and eventually assess the impact of covariates on those outcomes) [4, 12, 28–31]. By pointing out the treatment with the lowest probability of all types of events within a given time frame, it brings a perspective on the data that may be of interest for providers, payers, or policymakers who need to predict the burden on human and financial resources of clinical events on patients enrolled in the care system [16, 32].

### Cause-specific and subdistribution framework when survival competes safety

The results of our simulation study give a clear picture of the differences between both safety analysis settings. When analyzing safety data, prolonged survival in one group will mostly translate into a higher probability of adverse events in a subdistribution setting, where the risk is assessed by combining the hazards of all competing events within a single cumulative incidence function over the entire follow-up period. Our simulation shows that the results of the subdistribution analysis are always more pessimistic than the results of the cause-specific analysis. For the group with the longest survival and the safety advantage a change of the analysis setting translates in either a smaller advantage, no more advantage, a larger or even a newly found disadvantage.

However, the outcomes of neither the cause-specific nor the subdistribution settings are biased, they just answer different research questions. The subdistribution outcome reflects the effect of treatment on both safety and survival, with no possibility to differentiate between the two, while the cause-specific analysis reflects the effect of treatment on safety only.

When very serious adverse events are considered and longer living comes at the price of unbearable safety events, the outcome of the subdistribution analysis could be used to compare the safety profiles of both medicinal products. However, in most cases, prolonged survival is still very much desirable despite the occurrence of minor or manageable adverse events. In this case, the effect of survival present in the subdistribution outcome does not allow to interpret the safety profile of the intervention.

### Recommendations for clinical evaluations

As a general rule, we recommend, to first describe the competing risks as well as their expected impact on the analysis. When competing risks have been identified, competing risks analysis should be preferred to marginal analysis when the number of competing events in the study is at least equal to that of the event of interest [33], or when the absolute percentage of competing events is greater than 10% [13]. When competing risks analysis is indicated, we recommend a cause-specific setting, together with a justification of the choice of the competing events considered. This recommendation is in line with the suggestions made by the Committee for Medicinal Products for Human Use of the European Medicines Agency [7] in its Composite variable strategies.

The Cox proportional hazards model that is routinely presented in clinical study reports should remain the standard approach. The presentation of Kaplan-Meier estimates is also justified, although said to overestimate cumulative event probabilities [4, 6, 33, 34]. Kaplan-Meier in a cause-specific setting represents the absolute risk of having an event of interest, as if nothing else could happen before [29]. In comparison, Aalen-Johansen estimates the fraction of patients who will experience an event of interest within the given time frame, given the presence of other precluding events. The cause-specific setting therefore allows many more subjects to experience the event of interest. This explains the observation, also made in our simulation, that Kaplan-Meier estimates are systematically larger than those derived from the Aalen-Johansen method. Although this effect should be known and understood, we do not agree with the terminology commonly used in the literature that Kaplan-Meier “overestimates” the incidence of events. This wording implies that one setting delivers correct estimates and the other not, while it is in fact a matter of context.

As an alternative estimator to Kaplan-Meier for the same function, the Nelson-Aalen estimator could be considered [35]. Our simulation confirmed that it delivers the same information as the Kaplan-Meier estimator in comparative analysis, but its understanding is less straightforward. For this reason, the Nelson-Aalen estimator is less popular than the Kaplan-Meier estimator in time-to-event analysis since its first publication in the late 1950s [36]. As clinical study reports are also meant to be reviewed by non-statisticians such as medical experts and epidemiologists within the frame of regulatory activities and clinical evaluations, the well-known and commonly presented Kaplan-Meier curve should be favored. One might argue that there is no harm in presenting both, but we do not recommend it as a standard approach. Clinical reports usually contain large amounts of analyses, and the non-essential presentation of the Nelson-Aalen estimator for each endpoint, might cause most readers to feel overwhelmed.

### Limitations

In this study, we chose to keep the time to competing event constant in both study groups and across all simulated scenarios. It was therefore not possible to investigate further discrepancies between both settings on various times to occurrence of the competing events. It would be interesting to confirm that the conclusion of this work remains valid for a wide range of time to competing event. Also, the number of patients in both study groups was kept constant across the simulated scenarios. An interesting question to investigate would be how sample size influences the results. The size of the trial impacts the statistical significance that is the *p*-value and the breadth of the confidence interval of the estimates. Gaining deeper insights into the role of sample size is particularly interesting for the special case of rare disease and pediatric trials where only small numbers of eligible trial participants are available. Finally, event times were simulated with an exponential distribution. This simple, known, parametric distribution is widely used to simulate survival data to investigate the properties of the Cox Model [37]. It offers an easy control of the regression coefficients and has proportional hazards, which is advantageous for the implementation. However, it assumes that the baseline hazard function is constant, which is not always the case, especially in the setting of AEs, that tend to occur shortly after starting treatment and death, where some patients might be too sick to rescue when they enter the trial.

An exponential distribution was deemed sufficient for this application, where the focus was to compare methodological approaches rather than to perform a realistic description of various survival time data. However, more complex statistical approaches have been described [37–40] and it would be interesting to investigate how the simulation framework influences the results.

## Conclusions

When analyzing survival data in the presence of competing events, there is no absolute right or wrong when it comes to the choice between a cause-specific and a subdistribution setting. The decision rather depends on the research question at hand. We claim that the risk/benefit profile of a medication is better assessed in a cause-specific setting. The authorities in charge assess the effect of the intervention on the risk of experiencing adverse events. They need estimates of the instantaneous risk of adverse events while on treatment, as well as separate estimates of the effect of the intervention on the competing events. These requirements can be met in a cause-specific setting but not in a subdistribution setting where a single cumulative incidence function that includes all the risks in presence is estimated. The subdistribution setting may be relevant, however, if economic questions should be answered or when both events are similar in the clinical harm (e. g. Death and extremely serious adverse events that tremendously impact patients’ wellbeing and Quality of life). The Kaplan-Meier estimate of the survival function, or its complement, and the Cox proportional hazard model for comparative analysis should remain the standard approach in clinical study reports. In the presence of competing risks, they should be embedded in a cause-specific setting and the choice of the competing events in the analysis should be justified.

## Supplementary Information

**Additional file 1.** Non-parametric inference of survival data.

## Data Availability

The data that support the findings of this study are available from the corresponding author upon reasonable request.

## References

[1] Allignol A, Beyersmann J, Schmoor C (2016). Statistical issues in the analysis of adverse events in time-to-event data. Pharm Stat.

[2] Koller MT, Raatz H, Steyerberg EW, Wolbers M (2012). Competing risks and the clinical community: irrelevance or ignorance?. Stat Med.

[3] van Walraven C, McAlister FA (2016). Competing risk bias was common in Kaplan-Meier risk estimates published in prominent medical journals. J Clin Epidemiol.

[4] Schuster NA, Hoogendijk EO, Kok AAL, Twisk JWR, Heymans MW (2020). Ignoring competing events in the analysis of survival data may lead to biased results: a nonmathematical illustration of competing risk analysis. J Clin Epidemiol.

[5] Stegherr R, Beyersmann J, Jehl V, Rufibach K, Leverkus F, Schmoor C, Friede T (2021). Survival analysis for AdVerse events with VarYing follow-up times (SAVVY): rationale and statistical concept of a meta-analytic study. Biom J.

[6] Stegherr R, Schmoor C, Beyersmann J, Rufibach K, Jehl V, Brückner A (2021). Survival analysis for AdVerse events with VarYing follow-up times (SAVVY)-estimation of adverse event risks. Trials.

[7] European Medicines Agency. ICH E9 (R1) addendum on estimands and sensitivity analysis in clinical trials to the guideline on statistical principles for clinical trials EMA/CHMP/ICH/436221/2017. 2020. https://www.ema.europa.eu/documents/scientific-guideline/ich-e9-r1-addendum-estimands-sensitivity-analysis-clinical-trials-guideline-statistical-principles_en.pdf.

[8] Borgan Ø (1997). Three contributions to the encyclopedia of biostatistics: the Nelson-Aalen, Kaplan-Meier, and Aalen-Johansen.

[9] Therneau TM, Crowson C, Atkinson E. Multi-state models and competing risks. 2020. p. 1–29.

[10] Andersen PK, Geskus RB, de Witte T, Putter H (2012). Competing risks in epidemiology: possibilities and pitfalls. Int J Epidemiol.

[11] Geskus RB. Data analysis with competing risk and intermediate states. Chapman and Hall/CRC; 2016.

[12] Putter H, Fiocco M, Geskus RB (2007). Tutorial in biostatistics: competing risks and multi-state models. Stat Med.

[13] Fine JP, Gray RJ (1999). A proportional hazards model for the subdistribution of a competing risk. J Am Stat Assoc.

[14] Gray RJ (1988). A class of K-sample tests for comparing the cumulative incidence of a competing risk. Ann Stat.

[15] Edwards JK, Hester LL, Gokhale M, Lesko CR (2016). Methodologic issues when estimating risks in pharmacoepidemiology. Curr Epidemiol Rep.

[16] Klein JP, van Houwelingen HC, Ibrahim JG, Scheike TH. Handbook of survival analysis. 1st ed. 2013. (10.12.2013 ed.).

[17] Therneau TM, Grambsch PM (2000). Modeling survival data: extending the Cox model.

[18] Cox DR (1972). Regression models and life-tables. J R Stat Soc Ser B Methodol.

[19] Collet D (2015). Modelling survival data in medical research.

[20] Zwiener I, Blettner M, Hommel G (2011). Survival analysis part 15 of a series on evaluation of scientific publications. Dtsch Arztebl Int.

[21] Klein JP, Moeschberger ML (2003). Survival analysis - techniques for censored and truncated data.

[22] Rubin DB (1987). Multiple imputation for nonresponse in surveys.

[23] R.CoreTeam (2019). R: a language and environment for statistical computing.

[24] Therneau TM, Lumley T, Elizabeth A, Cynthia C. R package survival. 2021. https://github.com/therneau/survival.

[25] Gray B. R package cmprsk: competing risks regression. In: Subdistribution analysis of competing risks. 2022. https://cran.r-project.org/web/packages/cmprsk/index.html.

[26] van Buuren S, Groothuis-Oudshoorn K (2011). mice: multivariate imputation by chained equations in R. J Stat Softw.

[27] Wickham H (2016). ggplot2 elegant graphics for data analysis.

[28] Austin PC, Allignol A, Fine JP (2017). The number of primary events per variable affects estimation of the subdistribution hazard competing risks model. J Clin Epidemiol.

[29] Austin PC, Lee DS, Fine JP (2016). Introduction to the analysis of survival data in the presence of competing risks. Circulation.

[30] Lau B, Cole SR, Gange SJ (2009). Competing risk regression models for epidemiologic data. Am J Epidemiol.

[31] Van Der Pas S, Nelissen R, Fiocco M (2018). Different competing risks models for different questions may give similar results in arthroplasty registers in the presence of few events. Acta Orthop.

[32] Pepe MS, Mori M (1993). Kaplan-Meier, marginal or conditional probability curves in summarizing competing risks failure time data?. Stat Med.

[33] Berry SD, Ngo L, Samelson EJ, Kiel DP (2010). Competing risk of death: an important consideration in studies of older adults. J Am Geriatr Soc.

[34] Satagopan JM, Ben-Porat L, Berwick M, Robson M, Kutler D, Auerbach AD (2004). A note on competing risks in survival data analysis. Br J Cancer.

[35] Colosimo E, Ferreira F, Oliveira M, Sousa C (2002). Empirical comparisons between Kaplan-Meier and Nelson-Aalen survival function estimators. J Stat Comput Simul.

[36] Kaplan EL, Meier P (1958). Nonparametric estimation from incomplete observations. J Am Stat Assoc.

[37] Bender R, Augustin T, Blettner M (2005). Generating survival times to simulate Cox proportional hazards models. Stat Med.

[38] Beyersmann J, Allignol A, Schumacher M (2012). Competing risks and multistate models with R.

[39] Beyersmann J, Latouche A, Buchholz A, Schumacher M (2009). Simulating competing risks data in survival analysis. Stat Med.

[40] Wan F (2017). Simulating survival data with predefined censoring rates for proportional hazards models. Stat Med.

