# Supplementary material for: Safety analysis of new medications in clinical trials: a simulation study to assess the differences between cause-specific and subdistribution frameworks in the presence of competing events
Source: BMC Med Res Methodol. 2023 Jul 13;23:168. doi: 10.1186/s12874-023-01985-7 (PMC10339642; doi:10.1186/s12874-023-01985-7)
Supplement: Supplementary file 1 — Additional file 1. Non-parametric inference of survival data. [file 12874_2023_1985_MOESM1_ESM.pdf]

Additional file 1

Non-parametric inference of survival data

A.1 Cause-specific setting

The Kaplan-Meier estimator

The Kaplan-Meier estimator is a non-parametric estimator of the survival distribution function S(t) from censored data. It states the cumulative probability (or risk) of the event of interest not occurring by a certain time t , i. e. to remain event free until t. As a probability, the estimator takes its values in [0, 1] and is mathematically expressed as:

$$\hat{S}(t) = P(event\ free\ at\ t) = \prod_t (1 - \frac{e_t}{R_t}) \quad 3$$

where e_t is the number of events that occurred at t and R_t is the number of individuals at risk, that are alive and not censored just prior to each considered t (Borgan, 1997).

The complement of the survival function is the cumulative incidence function (or cumulative risk) of the event of interest occurring over [0, t]:

$$\hat{F}(t) = P(event\ at\ t_j) = 1 - \hat{S}(t) \quad 4$$

One advantage of the cumulative incidence function over the survival function is that it is visually easier to compare with the estimates that all refer to the occurrence of the event of interest.

When dealing with competing events in a cause-specific setting, the Kaplan-Meier estimator is used to separately estimate probabilities for each type of event, while treating the other competing events as censored in addition to those who are censored from loss to follow-up or withdrawal.

The log-rank test is used to compare Kaplan-Meier estimates and test for differences among the groups.

The Nelson-Aalen estimator

The Nelson-Aalen estimator is a non-parametric estimator of the cumulative hazard (or rate) function $\mathrm{h(t)}$. It states the instantaneous conditional hazard of the event of interest occurring within the (small) interval of time $\Delta_\mathrm{t} = \mathrm{T} + \mathrm{dt}$, assuming it has not occurred until then. The reference to Δ_t

rather than to t to estimate an instantaneous rate is necessary because the probability of a continuous random variable (here the time, t) being equal to a particular value is zero.

The estimate of the instantaneous hazard rate at t is mathematically expressed as:

$$\hat{h}(t) = \int_0^t P(event\ within\ \Delta_t | event\ free\ at\ t) = \lim_{dt \to 0} \frac{P(T + dt | T \geq t)}{dt}$$

where $P(T + dt | T \geq t)$ is the conditional probability of the event of interest occurring within Δ_t, provided that it has not happened before and dt is the width of the interval of time. Taking the limit as dt tends toward zero, gives the instantaneous rate of occurrence that is the risk of an event occurring at a given time. Although commonly referred to as so, $\mathrm{h(t)}$ is not a probability: once divided by dt, the probability in the numerator can become >1. The hazard rate therefore takes its values between [0, ∞].

The Nelson-Aalen estimator is the sum of the point-wise estimates $\mathrm{h(t)}$ and states the total (cumulative) amount of risk accumulated up to a certain time t. It is mathematically expressed as:

$$\widehat{\Lambda}(t) = \int_0^t h(t)\, dt = \sum_t (\frac{e_t}{R_t}) \qquad 5$$

where $\mathrm{e_t}$ is the number of events that occurred at t and R_t is the number of individuals at risk, that is alive and not censored just prior to t (Borgan, 1997).

When dealing with competing events in a cause-specific setting, the cumulative cause-specific hazard estimated by the Nelson-Aalen estimator can be assessed using only occurrences of the relevant event.

In the absence of competing risks, the hazard rate $h(t)$ is directly related to the risk of occurrence $F(t)$:

$$F_t = 1 - e^{-\int_0^t h_t dt}$$

which means that the Nelson-Aalen estimator relates to the Kaplan-Meier estimates:

$$\hat{S}(t) = e^{-\widehat{\Lambda}(t)}$$

However in a competing risks situation, even if both the rate and risk concepts generalize easily, their one-to-one relationship is lost because all the cause-specific hazards $h_i(t)$ of the competing events i∈ [1, n] are nedeed when computing each of the cumulative incidences $F_i(t)$.

A.2 Subdistribution setting

The Aalen-Johanson estimator

The Aalen-Johansen estimator is a multi-state, or “matrix” version of the Kaplan–Meier estimator (Borgan, 1997). It estimates the absolute (i. e. given all competing causes) risk of a given event at time t. It is an estimator of the cumulative incidence, or in this case “subdistribution”, function presented in Equation 4:

$$\widehat{F_i}(t) = \int_0^t S_i(t)\mathrm{h}_i(\mathrm{t})\,(t)dt = \sum_t \frac{e_{i,t}}{r_t}\hat{S}(t-1)$$

where i∈ [1, n] are the competing events considered, $\mathrm{e_t}$ is the number of events that occurred at t and R_t is the number of individuals at risk, that is alive and not censored just prior to t (Borgan, 1997). We recognize in $\frac{e_t}{R_t}$ an estimate of the cause-specific hazard for the event of interest at time t expressed in Equation 5 as the Nelson-Aalen estimator. $\hat{S}(t-1)$ is an estimate of the overall survival function at the previous time point. It can be estimated using the Kaplan-Meier estimator presented in Equation 3 (Edwards, Hester, Gokhale, & Lesko, 2016).

In the absence of competing risks, the Aalen-Johansen estimator reduces to the Kaplan-Meier estimator and the same values are estimated with both (Schuster, Hoogendijk, Kok, Twisk, & Heymans, 2020).

The cumulative incidence functions of events among different groups are compared using K-sample tests, as described in (Gray, 1988). The test statistic is analogous to the log-rank test used on the before mentioned Kaplan-Meier or Nelson-Aalen estimates (but based on comparing weighted averages of the hazard of the subdistribution function for the event of interest. It does not require the independent censoring assumption made by the log-rank test to be verified.
